# Machine Learning Models to Predict Future Frailty in Community-Dwelling Middle-Aged and Older Adults: The ELSA Cohort Study

**DOI:** 10.1093/gerona/glad127

**Published:** 2023-05-20

**Authors:** Daniel Eduardo da Cunha Leme, Cesar de Oliveira

**Affiliations:** Graduate Program in Gerontology, School of Medical Sciences, University of Campinas, Campinas, Brazil; Department of Epidemiology and Public Health, University College London, London, UK

**Keywords:** Artificial intelligence, Frailty, Outcome, Risk factors

## Abstract

**Background:**

Machine learning (ML) models can be used to predict future frailty in the community setting. However, outcome variables for epidemiologic data sets such as frailty usually have an imbalance between categories, that is, there are far fewer individuals classified as frail than as nonfrail, adversely affecting the performance of ML models when predicting the syndrome.

**Methods:**

A retrospective cohort study with participants (50 years or older) from the English Longitudinal Study of Ageing who were nonfrail at baseline (2008–2009) and reassessed for the frailty phenotype at 4-year follow-up (2012–2013). Social, clinical, and psychosocial baseline predictors were selected to predict frailty at follow-up in ML models (Logistic Regression, Random Forest [RF], Support Vector Machine, Neural Network, K-nearest neighbor, and Naive Bayes classifier).

**Results:**

Of all the 4 378 nonfrail participants at baseline, 347 became frail at follow-up. The proposed combined oversampling and undersampling method to adjust imbalanced data improved the performance of the models, and RF had the best performance, with areas under the receiver-operating characteristic curve and the precision-recall curve of 0.92 and 0.97, respectively, specificity of 0.83, sensitivity of 0.88, and balanced accuracy of 85.5% for balanced data. Age, chair-rise test, household wealth, balance problems, and self-rated health were the most important frailty predictors in most of the models trained with balanced data.

**Conclusions:**

ML proved useful in identifying individuals who became frail over time, and this result was made possible by balancing the data set. This study highlighted factors that may be useful in the early detection of frailty.

Frailty is a widely studied geriatric syndrome. It is characterized by social, clinical, and psychosocial stressors, and is associated with falls, hospitalization, institutionalization, disability, and mortality among older adults. It is also associated with high costs for health systems globally (1).

In a study of community-dwelling middle-aged and older adults in European countries, the prevalence of the frailty phenotype was 4.1% and 17.0%, respectively (2), and in a systematic review with meta-analysis, the global incidence of frailty was 13.6% in a mean of 3 years of follow-up (3). Given its clinical importance, many studies around the world have investigated the determinants of this syndrome. However, they employed traditional statistical methods such as regression analysis (4).

Network analysis, which is a more sophisticated statistical method, has also been used recently to determine the complex relationships between different factors and the frailty phenotype (5). However, the aforementioned statistical methods were used exclusively to estimate the effect of a set of independent or random variables on the variable of interest, that is, frailty.

Predictive models, such as those based on machine learning (ML), are intended primarily to make predictions that are as accurate as possible by learning from data. ML has become an important tool for identifying people with health vulnerabilities and can be used to help choose prevention and treatment strategies (6).

As statistical packages with different ML models, such as Logistic Regression (LR), Random Forest (RF), and Support Vector Machine (SVM), are now available, health researchers are using predictive models to identify older adults presenting with sarcopenia (7) and frailty (8). Although ML is useful in the field of health, it requires a balanced data set, that is, the outcome variable categories must have similar proportions. This is not the case in epidemiologic data sets, however, particularly when frailty is the outcome variable resulting in misidentification of this syndrome and predictive models with poor performance. Data sets can be balanced using methods that allow differences in the proportions of the outcome variable categories to be corrected (9), and it is relevant to the use of ML models as an accurate tool for early detection of frailty, decision making, and choosing the most appropriate health planning at a population level.

The present study, therefore, sought to compare the performance of ML models trained with epidemiologic data sets with and without balancing and to determine the most important variables when predicting future frailty in nonfrail participants in a population-based sample of community-dwelling middle-aged and older adults.

## Method

### Study Design and Participants

This study is a retrospective cohort study that used data from the English Longitudinal Study of Ageing (ELSA), which has been described previously (10). The initial sample consisted of 4 637 individuals aged 50 years or more who had been assessed according to the frailty phenotype (11) between 2008 and 2009 (baseline) and between 2012 and 2013 (follow-up). Of these individuals, 2 541 were not frail, 1 837 were pre-frail, and 259 were frail at baseline. The individuals who were frail at baseline were excluded, and 4 378 participants were included in the final analytical sample. Of all the nonfrail participants at baseline, 61% were reassessed for the phenotype. All the participants in the ELSA study signed a consent form, and the study was approved by The National Research Ethics Service (London Multicentre Research Ethics Committee [MREC/01/2/91]).

### Outcome Variable

At baseline and follow-up, frailty was defined according to the adapted model of the frailty phenotype (11), which consists of 5 components: (1) weakness: lowest quintile of grip strength, stratified by sex in each body mass index (BMI) quartile; (2) slow walking speed: lowest quintile of walking speed on 2.4-m distance course. The average time, in seconds, to complete 2 courses was stratified by sex and the average height, in centimeters, of the participants; (3) unintentional weight loss: defined as the loss of 5% of body weight in the previous wave of the study or by BMI < 18.5 kg/m in the wave of interest in this study (12); (4) low physical activity level: based on the intensity and frequency of physical activity performed by the participant, considering the intensity levels of vigorous, moderate, and mild physical activity, and the frequency of more than once per month, once per week, 1–3 times per week or never. Those who reported never performing moderate-intensity physical activity were classified as having a low physical activity level (13) and (5) exhaustion: defined as a positive response to any of the 2 questions: *Felt that everything I did was an effort in the last week* or *Could not get going in the last week* of the Center for Epidemiologic Studies—Depression scale (CES-D) (14). Individuals who scored in 3 or more of these components were classified as frail, whereas those who scored in only 1 or 2 components were classified as pre-frail and those who did not score in any component were classified as nonfrail. The outcome variable frailty has 5 categories: *nonfrail*, which included those participants who were nonfrail or pre-frail at baseline and whose status had not changed at follow-up and participants who changed from pre-frail at baseline to nonfrail at follow-up; and *frail*, which included individuals who were nonfrail or pre-frail at baseline and became frail at 4-year follow-up.

### Candidate Predictors

Baseline variables considered important for predicting physical frailty were selected (11,15,16). Sociodemographic variables were sex (male and female); marital status (has or does not have a partner); years of schooling (>13 years, 12–13 years, or 0–11 years); social class based on occupation (managerial and professional occupations, intermediate occupations, semiroutine and routine occupations, or other/never worked); and household wealth in quintiles (17).

Health and lifestyle variables were smoking (nonsmoker or former smoker/smoker); alcohol consumption (rarely/never, frequently, or daily); abdominal obesity (yes or no) (18); self-reported doctor-diagnosed cardiovascular disease (CVD), diabetes, chronic obstructive pulmonary disease, arthritis, osteoporosis, urinary incontinence, and brain disorders (yes or no); self-reported hearing and vision (very good, fair, or poor); self-reported fear of falling (yes or no); self-rated health (very good, fair, or poor); C-reactive protein level (high or low); pain status (no pain, mild, moderate, or severe) (19). Balance problems (never, sometimes, or very often/always) were reported by participants as the frequency of balance problems when they walked on a level surface (10). In the chair-rise test, participants were instructed to rise as fast as possible from a chair 5 times without using their arms, and the time taken to complete the test was recorded. Shorter times correspond to greater leg strength (10).

Psychosocial variables were elevated depressive symptoms (yes or no) defined by CES-D score of ≥4 (14); cognitive function based on the results of a cognitive screening test (a higher value in the test indicates better cognitive function) (20); self-reported memory (excellent/very good, good, or reasonable/poor); self-reported sleep quality (very good/good or bad/very bad); self-reported loneliness (almost never/never, sometimes, or very often), through self-report of how often the participant felt lonely (21); social network contact frequency (high, average, or low); and positive social support (high or low) (22).

### Statistical Analysis

Baseline categorical and numerical variables were compared with the chi-squared Pearson test and Mann–Whitney test, respectively, according to the frailty status at follow-up. In this stage, the data set without preprocessing was used. A significance level of *p* < .05 was used.

### Machine Learning Stages

Before the models were trained, the data were preprocessed. Variables with variance equal or close to 0 were removed. Of the 31 variables listed in the Method section, 3 were excluded (C-reactive protein level, self-reported fear of falling, and brain disorders). The numerical predictors did not have strong correlations with each other and were therefore not excluded (23).

The original data set was randomly divided into a training data set with 70% of the sample (2 822 nonfrail and 243 frail) and a test data set with 30% of the sample (1 209 nonfrail and 104 frail). The remaining preprocessing steps were carried out on the training data set. Some predictor variables had a percentage of missing data of less than 10% (Supplementary Figure 1). The data were, therefore, imputed with the RF method. The present study used variable standardization, a method by which the variables are redimensioned so that they have a mean of 0 and a standard deviation of 1; this method is less radical than normalization, which redimensions the variables so that they have values within the range 0–1. The choice of standardization was based on the types of predictors included in the models, namely categorical and numerical and thus with different amplitudes, and the fact that the chosen model was the K-nearest neighbor model (K-NN), which uses distance measures during training. Standardization of the data improved the performance of the models, justifying the use of this method. The method is available in the Caret package in the center and scale functions (23). We then selected the most important variables for predicting frailty by the Boruta method (24). Out of all the variables, 13 were considered important and were inserted in the ML model. The results and details of the variable selection procedure are given in Supplementary Figure 2.

In the last preprocessing stage, the data were balanced with the Synthetic Minority Oversampling Technique (SMOTE). This is a hybrid method that generates new samples of artificial or synthetic data based on information from the original samples, allowing a balance to be achieved between categories with more observations and those with fewer observations. It was decided to oversample the category with fewest observations and undersample the category with most observations simultaneously so that the data set was balanced with 729 frail individuals and 719 nonfrail individuals. Oversampling and undersampling were performed by adjusting the number of observations generated artificially in the frail category with the function perc.over = 200 and by selecting the number of observations in the category with the greatest number of participants by means of the function perc.under = 148. Specifically, the rule for the oversampling method was based on the calculation 200/100 = 2, so that twice the number of frail observations already in the sample were generated, giving a total of 729 observations in the frail category. The corresponding calculation for undersampling was 148/100 = 1.48, so that the number of nonfrail observations selected was 1.48 times the number of frail observations generated. As 486 frail observations were generated in addition to the 243 in the training data set, 719 nonfrail observations were selected in the nonfrail category so that the percentage of observations for each category was close to 50%, allowing a reasonable number of frail observations to be generated. The functions perc.over and perc.under can be found in the DMwR statistical package in RStudio version 1.1.463, Boston, MA (25). Supplementary Figure 3 shows in detail the distribution of the observations by category in the balanced and imbalanced training data sets.

After preprocessing, the imbalanced and balanced data sets were divided into 5 random parts in the cross-validation test, which was repeated 10 times in order to randomize the training and validation data and improve model performance. In this stage, the models were manually adjusted during training and selected based on the best value of the receiver-operating characteristic (ROC), specificity, and sensitivity. The values of these metrics achieved with each model selected for testing and the parameters used are shown in Supplementary Table 1. The following 6 ML models were trained: LR, RF, SVM, Neural Network (NNET), K-NN, and Naive Bayes classifier (NB) (23).

**Figure 1.**
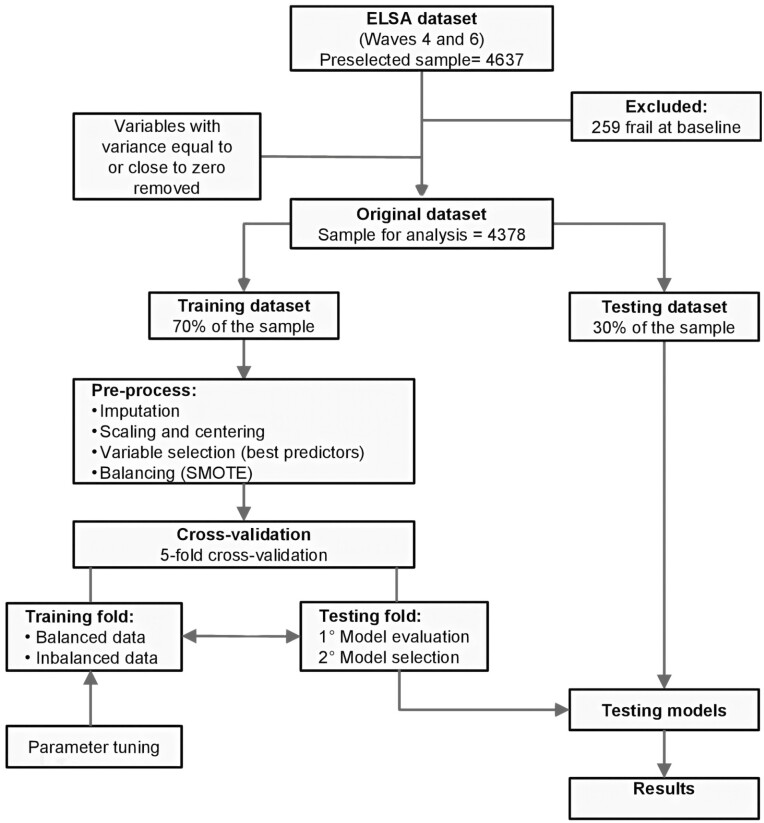
Flowchart of the machine learning stages in this study. ^a^The ELSA data set (Waves 4 and 6) was preselected and the frail participants were excluded from the present study. The preprocessing was performed on the training data set, which was used to select the best models that were tested with the testing data set. ELSA = English Longitudinal Study of Aging; SMOTE = Synthetic Minority Oversampling Technique.

Finally, the test data set was used to evaluate the performance of the models trained on the imbalanced and balanced data sets to assess how well these models fitted the data in the test data set, which was not used in the training stage. The ROC and precision-recall curves and the metrics area under the ROC curve (AUC), accuracy, balanced accuracy, specificity, sensitivity, positive predictive value (PPV), and negative predictive value were used to assess the performance of the 6 models. The Youden index was used to determine the optimal threshold for the prediction of frailty by optimizing specificity and sensitivity. RStudio version 1.1.463 was used for all the data analyses. The Caret package was used in the ML stages (23). Figure 1 shows the ML stages in this study in a flowchart.

## Results

Of all the nonfrail individuals at baseline, 61% were reassessed for the frailty phenotype at 4 years of follow-up. At baseline, the median age was 65 years (interquartile range, 60.0–71.0 years) and most participants were women (55.0%). In all, 347 (7.9%) individuals became frail and those who became frail were older, less wealthy, and frequently or always reported balance problems at baseline. They also took longer to complete the chair-rise test (Table 1).

**Table 1.** Baseline Variables Selected for Machine Learning by Frailty Status at Follow-Up in the Data Set Without Preprocessing

		Frailty		
	Total^†^	Nonfrail	Frail	
Variables	(*N* = 4 378)	(*N* = 4 031)	(*N* = 347)	*p* Value
Age (range, 50–90 y), median (IQR), y	65.0 (60.0–71.0)	64.0 (60.0–70.0)	73.0 (66.0–80.0)	<.001^*^
Sex, no. (%)				0.272
Female	2 369 (55.0)	2 171 (53.9)	198 (57.1)	
Male	2 009 (45.0)	1 860 (46.1)	149 (42.9)	
Household wealth, no. (%)				<.001^*^
Fifth quintile (highest quintile)	1 154 (26.8)	1 110 (28.0)	44 (12.8)	
Fourth quintile	1 008 (23.4)	943 (23.8)	65 (18.9)	
Third quintile	903 (21.0)	827 (20.9)	76 (22.1)	
Second quintile	739 (17.2)	676 (17.1)	63 (18.3)	
First quintile (lowest quintile)	490 (11.4)	395 (10.0)	95 (27.7)	
Alcohol consumption, no. (%)				<.001^*^
Rarely or never	651 (16.0)	550 (14.6)	101 (32.4)	
Frequently	1 776 (43.7)	1 648 (43.9)	128 (41.1)	
Daily	1 631 (40.1)	1 549 (41.3)	82 (26.3)	
CVD, no. (%)				<.001^*^
No	3 738 (85.9)	3 496 (87.2)	242 (70.5)	
Yes	610 (14.0)	509 (12.7)	101 (29.4)	
Diabetes, no. (%)				<.001^*^
No	4 006 (92.1)	3 721 (92.9)	285 (83.0)	
Yes	342 (7.8)	284 (7.0)	58 (16.9)	
Pain status, no. (%)				<.001^*^
No pain	2 831 (64.7)	2 686 (66.6)	145 (41.8)	
Mild pain	548 (12.5)	512 (12.7)	36 (10.4)	
Moderate pain	778 (17.8)	668 (16.5)	110 (31.7)	
Severe pain	218 (4.9)	162 (4.0)	56 (16.1)	
Balance problems, no. (%)				<.001^*^
Never	3 619 (82.9)	3 446 (85.7)	173 (50.0)	
Sometimes	581 (13.3)	466 (11.5)	115 (33.2)	
Very often/always	165 (3.7)	107 (2.6)	58 (16.7)	
Chair rise (range, 5.0–40.7 s), median (IQR), s	10.5 (8.6–12.7)	10.4 (8.5–12.4)	13.8 (11.1–16.0)	<.001^*^
Self-rated health, no. (%)				<.001^*^
Very good	3 627 (82.9)	3 453 (85.6)	174 (50.1)	
Fair	645 (14.7)	515 (12.8)	130 (37.5)	
Poor	106 (2.4)	63 (1.6)	43 (12.4)	
Vision status, no. (%)				<.001^*^
Excellent/very good	2 337 (53.4)	2 202 (54.6)	135 (38.9)	
Good	1 648 (37.6)	1 510 (37.5)	138 (39.8)	
Fair/poor	393 (9.0)	319 (7.9)	74 (21.3)	
Depression, no. (%)				<.001^*^
No	3 979 (91.3)	3 702 (92.3)	277 (79.8)	
Yes	378 (8.7)	308 (7.7)	70 (20.1)	
Sleep quality, no. (%)				<.001^*^
Very good/good	3 586 (82.0)	3 335 (82.8)	251 (72.3)	
Very bad/bad	789 (18.0)	693 (17.2)	96 (27.7)	
Loneliness, no. (%)				<.001^*^
Did not experience loneliness	2 975 (72.9)	2 787 (74.1)	188 (59.1)	
Experienced loneliness	1 104 (27.1)	974 (24.9)	130 (40.8)	

*Notes*: CVD = cardiovascular disease; Chair rise = chair-rise test; IQR = interquartile range.

^†^Because of missing data, the frequencies may not add up to the values at the top of the columns and the percentages may not add up to 100%.

^*^Statistical difference between the nonfrail and frail groups (*p* < .05).

**Figure 2.**
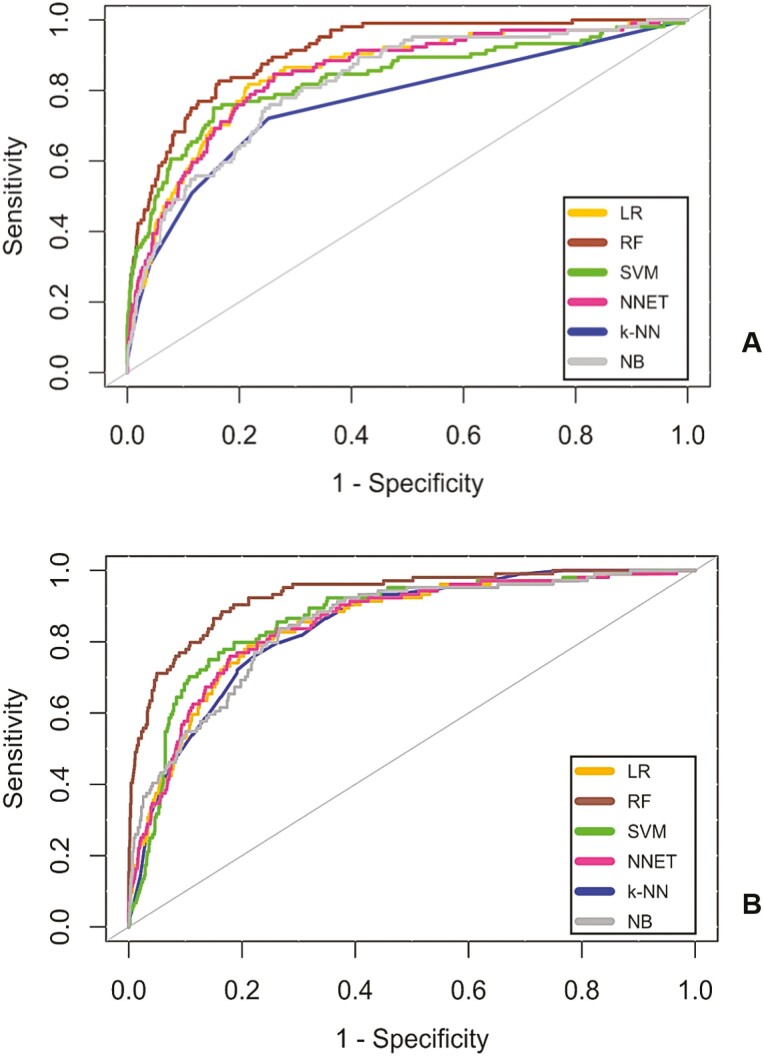
Receiver-operating characteristic curves of the models trained with imbalanced (A) and balanced (B) data sets. K-NN = K-nearest neighbor; LR = Logistic Regression; NB = Naive Bayes classifier; NNET = Neural Network; RF = Random Forest; SVM = Support Vector Machine.

Figure 2 shows the ROC curves for the 6 ML models trained with imbalanced (A) and balanced (B) data sets. The performance of most models improved when the balanced data set was used, and the RF model had the best performance after this procedure (Figure 2). Supplementary Figure 4 shows the PR curves with the respective AUC values for the 6 ML models trained with imbalanced and balanced data. There was an improvement in the performance of all the models, especially RF, which yielded the best PR curve and the best PRAUC (0.97) after balancing (Supplementary Figure 4).

Table 2 shows the performance of the ML models trained with imbalanced and balanced data sets based on different metrics. All the models yielded better values for the metric accuracy when they were trained with imbalanced data. However, all the models yielded better values for balanced accuracy, sensitivity, and PPV when they were trained with the balanced data set, and RF had the best values for AUC (0.92; 95% confidence interval: 0.88–0.93), specificity (0.83), sensitivity (0.88), and PPV (0.56) compared with all the other models trained with balanced data (Table 2).

**Table 2.** Performance of the Models Trained With Imbalanced and Balanced Data Sets Based on Different Metrics

	Accuracy %	Balanced Accuracy %	AUC (95% CI)	Threshold^*^	Specificity	Sensitivity	PPV	NPV
Imbalanced Data Model								
LR	90.01	75.2	0.84 (0.81–0.88)	0.231	0.78	0.72	0.37	0.86
RF	91.78	80.4	0.90 (0.88–0.94)	0.250	0.83	0.77	0.45	0.90
SVM	90.69	72.3	0.83 (0.78–0.88)	0.103	0.82	0.62	0.33	0.97
NNET	91.09	72.4	0.85 (0.80–0.88)	0.301	0.74	0.70	0.37	0.89
K-NN	90.78	69.5	0.75 (0.72–0.81)	0.201	0.73	0.65	0.34	0.89
NB	88.94	74.8	0.81 (0.77–0.86)	0.142	0.74	0.75	0.29	0.88
Balanced Data Model								
LR	88.23	81.0	0.85 (0.81–0.88)	0.483	0.79	0.83	0.42	0.88
RF	89.90	85.5	0.92 (0.88–0.93)	0.449	0.83	0.88	0.56	0.89
SVM	81.97	78.1	0.89 (0.86–0.93)	0.236	0.77	0.79	0.38	0.83
NNET	87.86	82.0	0.87 (0.83–0.89)	0.552	0.79	0.85	0.52	0.85
K-NN	85.25	80.3	0.84 (0.80–0.87)	0.574	0.76	0.84	0.36	0.90
NB	83.94	80.2	0.85 (0.81–0.88)	0.245	0.79	0.81	0.49	0.91

*Notes*: AUC= area under the receiver-operating characteristic curve; CI = confidence interval; K-NN= K-nearest neighbor; LR= Logistic Regression; NB= Naive Bayes classifier; NNET= Neural Network; NPV= Negative Predictive Value; PPV= Positive Predictive Value; RF= Random Forest; SVM= Support Vector Machine.

^*^The Youden index was used to determine the optimal threshold for the prediction of frailty by optimizing specificity and sensitivity.

**Figure 3.**
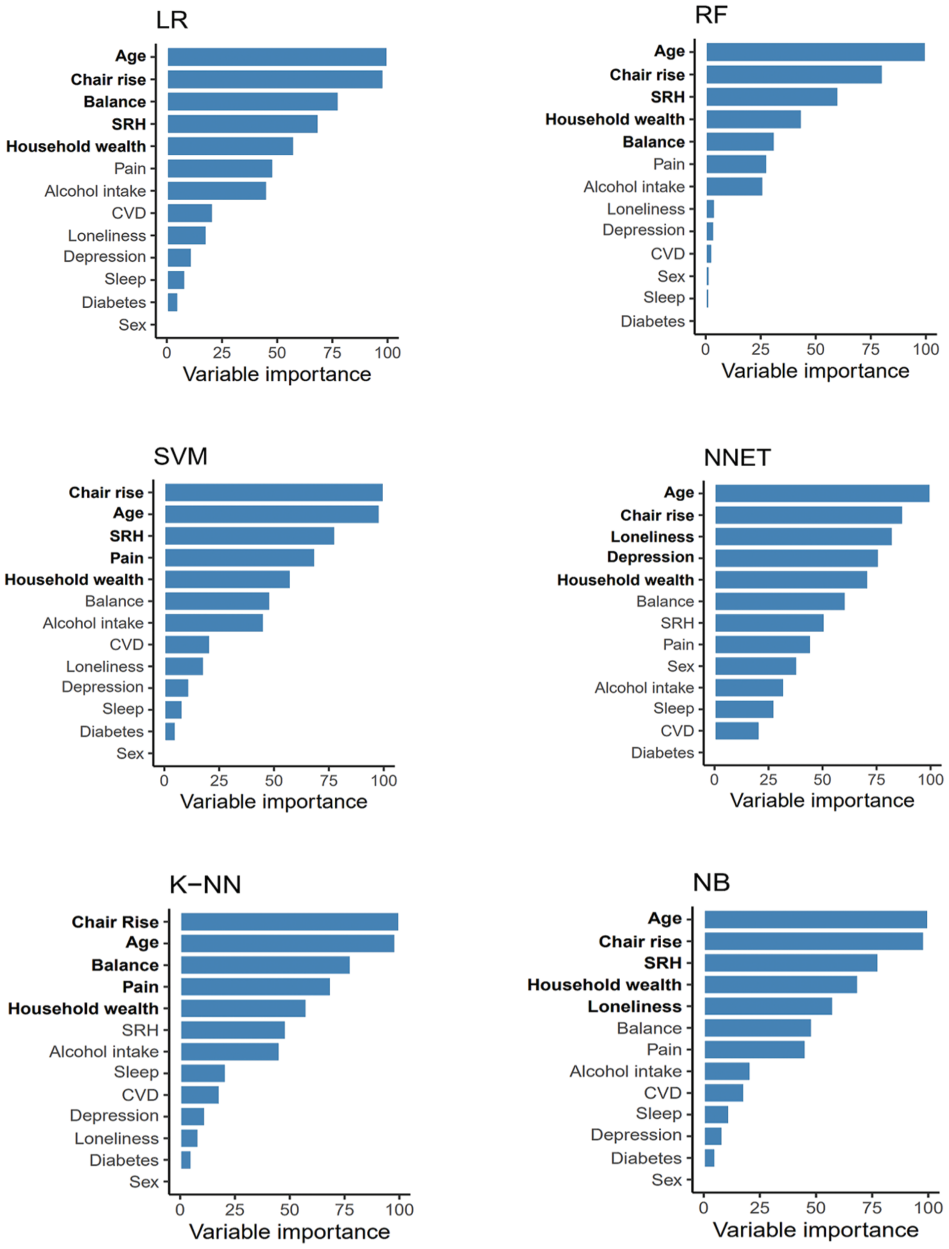
Importance of the variables in the machine learning models trained with a balanced data set. Balance = balance problems; CVD = cardiovascular disease; Chair rise = chair-rise test; K-NN= K-nearest neighbor; LR = Logistic Regression; NB = Naive Bayes classifier; NNET= Neural Network; RF = Random Forest; SVM= Support Vector Machine; Pain = pain status; SRH = self-rated health; Vision= vision status. ^a^The 5 most important variables are shown in bold.

Figure 3 shows the importance of each variable in the models trained with the balanced data set. Age, chair-rise test, and household wealth were more important in all the models, followed by self-reported health, which was among the most important variables in 4 models (LR, RF, SVM, and NB), and balance problems, which was important in 3 models (LR, RF, and K-NN). NNET was the only model in which depression and loneliness were important variables (Figure 3). Furthermore, to verify the impact of the aforementioned variables on frailty, partial dependence plots were estimated for each ML model trained with the balanced data set. More specifically, during the analysis of 6 different ML models, the probability of frailty increased with older age, higher scores on the chair-rise test, balance problems when self-reported as occurring very often or always, lower household wealth quintile, poor self-reported health, and loneliness. Supplementary Figure 5 shows in detail how each variable considered important in the ML models affects the syndrome.

## Discussion

This study compared the performance of different ML models trained with epidemiologic data sets with and without balancing and identified the most important variables when predicting future frailty in nonfrail middle-aged and older adults. Balancing led to an improvement in the performance of the models, especially in sensitivity, and RF had the best values of AUC, balanced accuracy, specificity, sensitivity, and PPV after balancing. In addition, age, chair-rise test, and household wealth were the most important variables for predicting frailty in all models, followed by self-reported health and balance problems in the balanced data set.

The models trained in this study are widely used in studies in the field of health. LR is a classical model that does not require parameter fitting, as a result of which it is frequently used in studies investigating common conditions among older adults, such as falls, cognitive deficit, and mortality (26–28). Despite the popularity of LR, the RF, SVM, NNET, K-NN, and BN models have gained increasing attention in the last years and have recently been used to study the predictors of frailty (8).

The RF model stood out among all the others. This model is used in classification and regression problems, which belong to what are known as *ensemble methods*, that is, methods that can be used to construct multiple models and combine them to reach a final result. The RF model is robust and has been widely used in the field of health in recent years because of its good predictive capacity (29).

Studies differ as to the best model for predicting frailty. A longitudinal study used RF, LR, SVM, and Lasso-penalized logistic regression (Lasso-LR) to predict the frailty phenotype in 4 668 older adults. To solve the imbalanced data problem, the authors applied the oversampling strategy to increase the number of observations in the minority category. LR performed better after balancing (AUC = 0.71). Age, sex, time since last discharge from hospital, and a higher cumulative score in terms of deficits in sensory, musculoskeletal, cardiovascular, nervous, urinary, and respiratory systems were the most important variables in the models (30).

Another longitudinal study used NB, SVM, and linear discriminant analysis to predict the frailty phenotype in 474 older adults by means of 284 predictors, including self-reported chronic diseases, medication, depression, score on the Mini-Mental State Examination, and mobility score. The authors applied the *k*-fold stratified cross-validation strategy to have the same number of frail and nonfrail observations in each created fold, and SVM had the best performance (AUC = 0.77) (31).

Although these previous studies included data set balancing in preprocessing, the authors used different data-level and algorithm-level methods for imbalanced data, such as oversampling and stratified cross-validation strategies, respectively, obtaining ML models with lower performance than our study. It must be emphasized that oversampling and stratified cross-validation strategies are frequently used when the data are initially imbalanced. However, the hybrid method SMOTE can be integrated with advanced techniques in ML and can get better results (32,33). In addition, it is also important to highlight that the previous studies used different predictors, models and sample sizes, and the methods were not standardized, making comparison with our results difficult.

In the present study, data balancing improved the ROC and PR curves, AUC, PPV, and especially sensitivity of the models. In imbalanced data sets, ML models ignore the category with the least number of observations and do not identify individuals that belong to it. Models trained with imbalanced data sets can, therefore, have inferior performance (9). Indeed, the frail category had a much lower percentage of observations, and the models trained with the imbalanced data set generally had a lower sensitivity, which in this study corresponds to the proportion of frail individuals identified correctly by the models (true positives), and a lower PPV, which corresponds to the proportion of cases classified as frail that were in fact frail. Concerning the PPV metric, when the data were initially imbalanced, our analyses showed values between 0.29 and 0.45. This corroborates the literature, which emphasizes that the extreme imbalance between categories of the outcome variable can affect the value of this metric (34). Although the PPV values were initially very low when the data were balanced, the RF model achieved a PPV value of 0.56, meaning that in our model, 1 out of 2 people may develop frailty in the future, which is clinically useful.

A recent study used SVM, K-NN, NB, NNET, RF, Elastic Net Logistic Regression, classification, and regression tree (CaRT) and eXtreme Gradient Boosting (XGBoost) to predict frailty in older adults and found that the performance of the models did not improve after balancing. According to the authors, the way the data were balanced may have affected the performance of the models. Only the frail category was oversampled. The fact that the nonfrail category was not undersampled meant that the computer had to increase the amount of oversampling to balance the categories. In this way, more frail individuals were introduced into the sample, increasing the possibility of these new observations not, in fact, corresponding to frail individuals. In balanced data, the XGBoost had the best performance with AUC of 80.5% and specificity of 93.2%; however, the sensitivity (37.9%) was lower than our study (8).

It has been shown in the literature that a combination of undersampling and oversampling can improve the performance of ML models (25). Therefore, in the present study, we sought to perform data balancing using undersampling and oversampling in such a way that the number of observations produced in the frail category was not increased, allowing more older adults that were actually frail to be identified. This was mainly confirmed by the increase in sensitivity after balancing. Models that have a high sensitivity are concerned, can be effective in identifying and diagnosing health problems (35).

With the imbalanced data set, all the models had better accuracy. Imbalanced data sets can have high accuracy, but this metric considers the correct predictions in both categories divided by the total number of predictions made (36). In the present study, the accuracy of 91.78% for RF observed with the imbalanced data set is related to the many nonfrail individuals identified and the smaller number of frail individuals identified. The use of accuracy to predict the frailty phenotype is, therefore, not recommended although this is a metric that is still used in epidemiologic studies with imbalanced data sets (8,9). Thus, balanced accuracy was used which is the arithmetic mean of sensitivity and specificity. Balanced accuracy has been employed in several areas including health sciences, to evaluate the performance of ML models trained on imbalanced data (37).

Only 13 variables were selected here for the training stage. Although ML can handle a much larger number of predictors, before including variables in a model consideration should be given to their clinical usefulness, the difficulty involved in obtaining them from the data set, and whether certain variables can improve the performance of the model (26). These issues were considered in the present study.

Age and household wealth were among the most important variables in the models trained with a balanced data set, corroborating previous studies which showed that these variables were associated with frailty and that social vulnerabilities are associated with worse health in older adults (1,16,38).

The chair-rise test was also an important variable in the models. This test detects functional decline, measures leg strength, and is one of the measures used to diagnose sarcopenia (39). In older adults, there is a very close relationship between leg strength and handgrip strength, which is a component of the frailty phenotype (40), and recently, the literature has shown an important association between the rise from a chair as a component of the SARC-F questionnaire and the frailty phenotype in older adults (41). This partly explains our finding. Although this test is suitable for identifying vulnerabilities and affordable in clinical practice, as it is easy to use compared with other clinical tests and can be used in a clinical or home setting, little is known about the importance of this variable in predictive models with multiple determinants of frailty phenotype in community-dwelling middle-aged and older adults.

Self-reported balance problems while walking were an important predictor of frailty. Unlike this study, an earlier study used static balance in statistical models and found that this variable was not associated with frailty in community-dwelling older adults (42). Problems with dynamic balance are associated with aging in different physiological systems, such as the muscular, nervous, sensory, and vestibular systems, and good communication between these systems is required to maintain balance and postural control. Balance is, therefore, a physical and cognitive health parameter (43). Furthermore, impaired dynamic balance has been shown to be very closely associated with falls in older adults (44), and an earlier study found that altered results in a test known as dynamic posturography during a dual-task protocol were associated with pre-frailty and frailty in older adults (45).

In the models trained in the present study, the variable self-rated health was found to be an important predictor of the frailty phenotype. Self-rated health is a subjective indicator of health status that includes aspects of mental and physical health, is easy to apply, and can detect vulnerabilities early (46). Self-rated health problems are associated with negative outcomes in older adults such as mortality and disability. A recent study with older adults seen at the primary health care level found that the variable self-rated health detected the frailty phenotype with a sensitivity of 62.5% and specificity of 93.6% (47). The results reported in the present study for the clinical predictors’ chair-rise test, balance problems, and self-rated health can help to guide health planning and the prevention of frailty, particularly as far as protocols for muscle-strengthening exercises, improvements in static and dynamic balance, posture control, and the management of clinical and subclinical conditions by health care professionals from different areas are concerned.

This study has limitations. First, the frailty phenotype is a predominantly physical measure. Although studies have shown a relationship between social and psychosocial factors and the frailty phenotype, other instruments that consider multidimensional aspects can yield different results as they capture social vulnerabilities among older adults (15,16). However, it should be stressed that in most of the models, household wealth was an important variable, and in the NNET model, which also had a good performance, loneliness was among the most important variables. Second, most of the variables used were self-reported and therefore subject to measurement errors, which can result in a bias in the analyses. Although this is an important issue in relation to the quality of the data used to train ML algorithms, self-reported variables are very common in epidemiologic studies and bias in analyses related to the use of these predictors is difficult to avoid. Another limitation is the loss of observations during the follow-up period, which may have had an impact on the results. However, this occurs frequently in longitudinal epidemiologic studies, particularly those involving older adults. Finally, individuals with disabilities were included at baseline. However, although sample selection allowed people with worse health to be included at the start of the study, this does not reduce the importance of the results as removing older adults with worse health status could reduce the sample even more and adversely affect the performance of the models.

A positive aspect of the study is that we used robust ML models in the prediction of future frailty. These models were also compared with established metrics within the ML literature. Another strength of this work is that the predictors highlighted in the existing frailty literature and data sets were considered, allowing for comparisons to be made with, and the ML models trained in the present study to be validated against, other data sets with a population of similar older adults. Lastly, although many researchers in ML use large data sets, as well as training models with many predictors, this study demonstrated that it is possible to apply ML in data sets with an acceptable number of observations and predictors, especially when the classes are initially imbalanced.

## Conclusion

In the present study, fitting the data by balancing the frailty variable categories proved effective. A comparison of the performance of the models trained with imbalanced data and with balanced data suggests that the ML approach was useful in identifying individuals who became frail over time. This result was made possible by the use of undersampling and oversampling techniques at the same time. Furthermore, this study has highlighted factors that can be useful for early detection of frailty in community-dwelling middle-aged and older adults; these factors can be found in most data sets and can be determined in clinical settings by means of muscle weakness tests and self-reported health and dynamic balance problems. Identification at the population level of individuals who may become frail can help with decision making in connection with prevention and management of the syndrome in order to avoid negative outcomes and reduce health-system costs.

## Supplementary Material

glad127_suppl_Supplementary_Material
