# Supplementary material for: Machine Learning Models to Predict Future Frailty in Community-Dwelling Middle-Aged and Older Adults: The ELSA Cohort Study
Source: J Gerontol A Biol Sci Med Sci. 2023 May 20;78(11):2176–84. doi: 10.1093/gerona/glad127 (PMC10613015; doi:10.1093/gerona/glad127)
Supplement: glad127_suppl_Supplementary_Material [file glad127_suppl_supplementary_material.docx]

**Supplementary Material**

**Machine Learning Models to Predict Future Frailty in Community-dwelling Middle-aged and Older Adults: the ELSA cohort study**

**Contents list**

**Supplementary Figure 1.** Frequency and percentage of absent data among the predictor variables in the training dataset

**Supplementary Figure 2.** Results of variable selection by the Boruta method

**Supplementary Figure 3.** Number of observations in each of the categories of the frailty variable in the imbalanced (A) and balanced (B) training datasets.

**Supplementary Figure 4.** Precision Recall curves of the models trained with imbalanced (A) and balanced (B) datasets.

**Supplementary Figure 5.** Partial dependence plots with numerical (A) and categorical (B) frailty predictors by ML models trained with the balanced dataset.

**Supplementary Table 1.** Values of ROC, specificity and sensitivity achieved with each model selected for testing and parameters used

Supplementary Figure 1 shows the frequency and cumulative percentage of missing data among the frailty predictor variables in the training dataset. Of all the variables for which data were missing, the chair-rise test had the greatest number of missing data (8.5%), followed by alcohol consumption (7.5%), loneliness (7.1%) and social network contact frequency (6.2%). Missing data corresponded to less than 10% for each variable, i.e., an acceptable value for the data imputation procedure. If this figure had been higher than 20% for any variable, the variable in question could have been removed from the data-analysis process unless it was known to be important (1) (Supplementary Figure 1).

**Supplementary Figure 1.** Frequency and percentage of missing data among the predictor variables in the training dataset

Note: Chair rise= chair-rise test; Abdominal obes= abdominal obesity; CVD= cardiovascular disease; COPD= chronic obstructive pulmonary disease; Occupational= social class based on occupation; Balance= balance problems; Memory= self-rated memory; Pain= pain status; and Sleep= quality of sleep.

^a^Only variables with missing data are shown in the figure

Supplementary figure 2 shows the result of selection by the Boruta method of variables that are important for predicting frailty. The Boruta method randomly creates artificial variables in the dataset, which are called shadows. Next, the Random Forest (RF) model was trained with the artificial and real variables. At each iteration of RF, the method compares the value of the importance of the real variables with the value of the importance of the shadow variables. If each real variable receives a greater measure than a shadow variable, it is considered important in the model [2]. The y-axis of the figure below represents the value in means of the measure of importance of the variables, which is called mean decrease accuracy. In the present study this measure varies from 0 to 30. Variables with the greatest value of mean decrease accuracy are represented by green box-plots and are considered important. Variables with a lower value of this measure are represented by red box-plots and are considered unimportant and should be removed from the ML models. When the Boruta method is unable to identify whether a variable is important, the variable is represented by a yellow box-plot and is considered tentative. In this stage, 13 variables were considered important: age, self-rated health, sleep problems, chair-rise test, alcohol consumption, depression, household wealth, diabetes, cardiovascular disease, sleep quality, pain status, loneliness and sex (Supplementary Figure 2).

**Supplementary figure 2.** Result of variable selection by the Boruta method

Note: COPD= chronic obstructive pulmonary disease; Pos. Support= positive social support; Occupational= social class based on occupation; Network freq= social network contact frequency; Abdominal obes= abdominal obesity; Marital st= marital status; Hearing= hearing status; Memory= self-rated memory; Cognition= cognition function; Vision= vision status; Pain= pain status; Sleep= sleep quality; CVD= cardiovascular disease; Chair rise= chair-rise test; Balance= balance problems; and SHR= self-rated health.

^a^Boruta method applied only to the training dataset

Supplementary Figure 3 shows the number of observations in each of the categories of the frailty outcome variable in the imbalanced (A) and balanced (B) training datasets. The SMOTE function was used with the command perc.over=200. This corresponds to oversampling, which adjusts the number of observations generated artificially in the frail category by means of the calculation 200/100=2, i.e., twice as many frail observations were generated in the sample (n=486) as there were in the training dataset (n=243), giving a total of 729 observations in the frail category. Similarly, the perc.under=148 command, which corresponds to undersampling, was used to adjust the number of non-frail observations selected from the category with the greatest number of observations. Based on the calculation 148/100=1.48, the number of non-frail observations selected was 1.48 times the number of frail observations generated (n=486), giving a total of 719 non-frail observations so that the two categories of the frailty variable were balanced, with approximately 50% of observations in each category (Supplementary Figure 3).


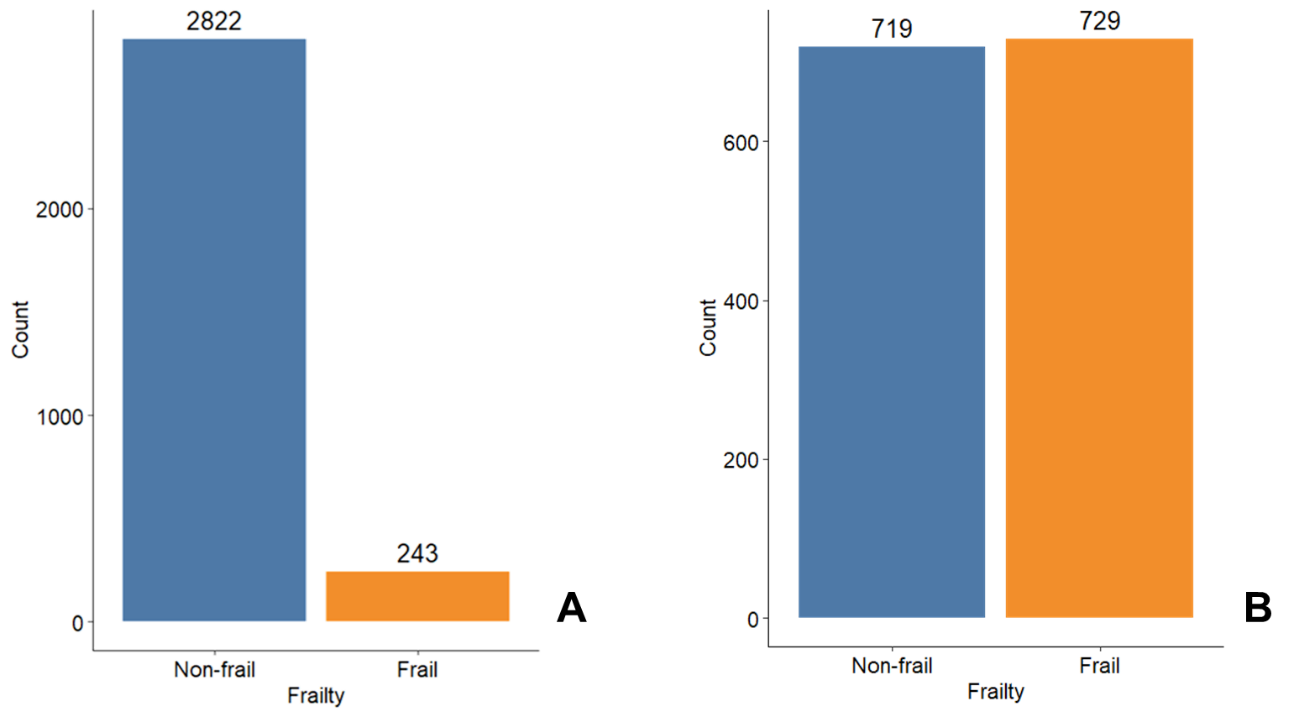


**Supplementary Figure 3.** Number of observations in each of the categories of the frailty variable in the imbalanced (A) and balanced (B) training datasets.


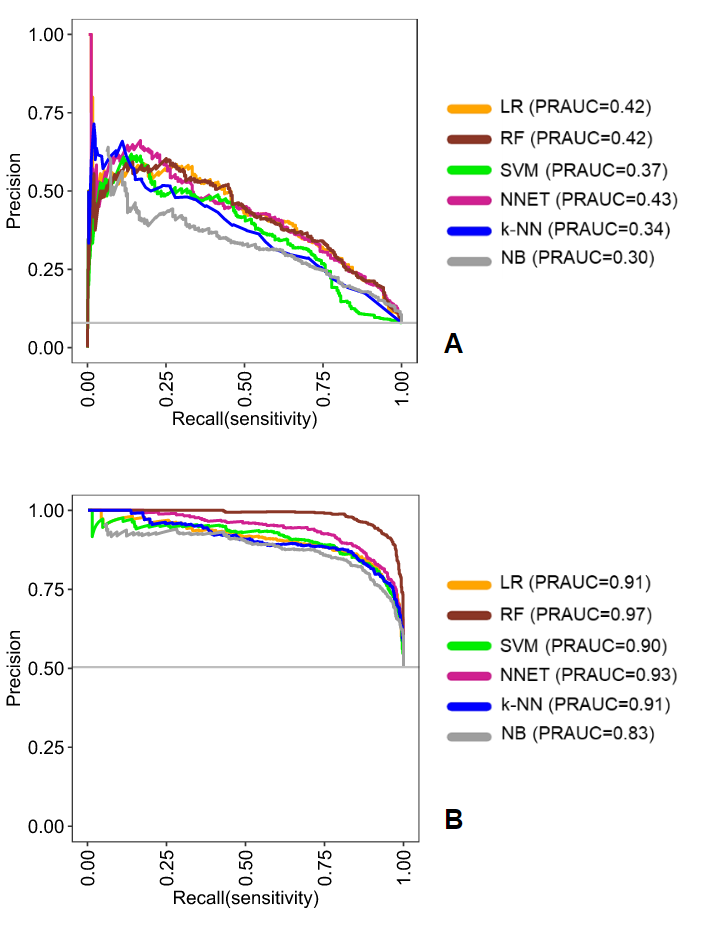


**Supplementary Figure 4.** Precision Recall curves of the models trained with imbalanced (A) and balanced (B) datasets.

Note: LR = Logistic Regression; RF = Random Forest; SVM = Support Vector Machine; NNET = Neural Network; K-NN = K-Nearest Neighbour; NB = Naive Bayes classifier; PRAUC = Precision Recall area under the curve.

^a^The horizontal gray line in the graphs represents the baseline value of the PR curves for imbalanced (A) and balanced (B) data. The baseline of 0.50 for the PR curves with balanced data (B) corresponds to balanced frail and non-frail categories.

Supplementary Figure 5 shows the PDPs with numerical (A) and categorical (B) frailty predictors considered important in ML models trained with the balanced dataset. The likelihood of frailty increased with older age, as well as higher scores on the chair-raise test. Moreover, the predictive capacity of these two numerical variables was greater in the RF model. Regarding categorical predictors, the likelihood of frailty increased with balance problems when self-reported as occurring very often or always, depression, lower levels of household wealth, loneliness, in addition to poor self-reported health. In the RF model, the predictive capacities of the variables relating to balance problems and poor self-reported health were relatively higher compared to other models (Supplementary Figure 5).


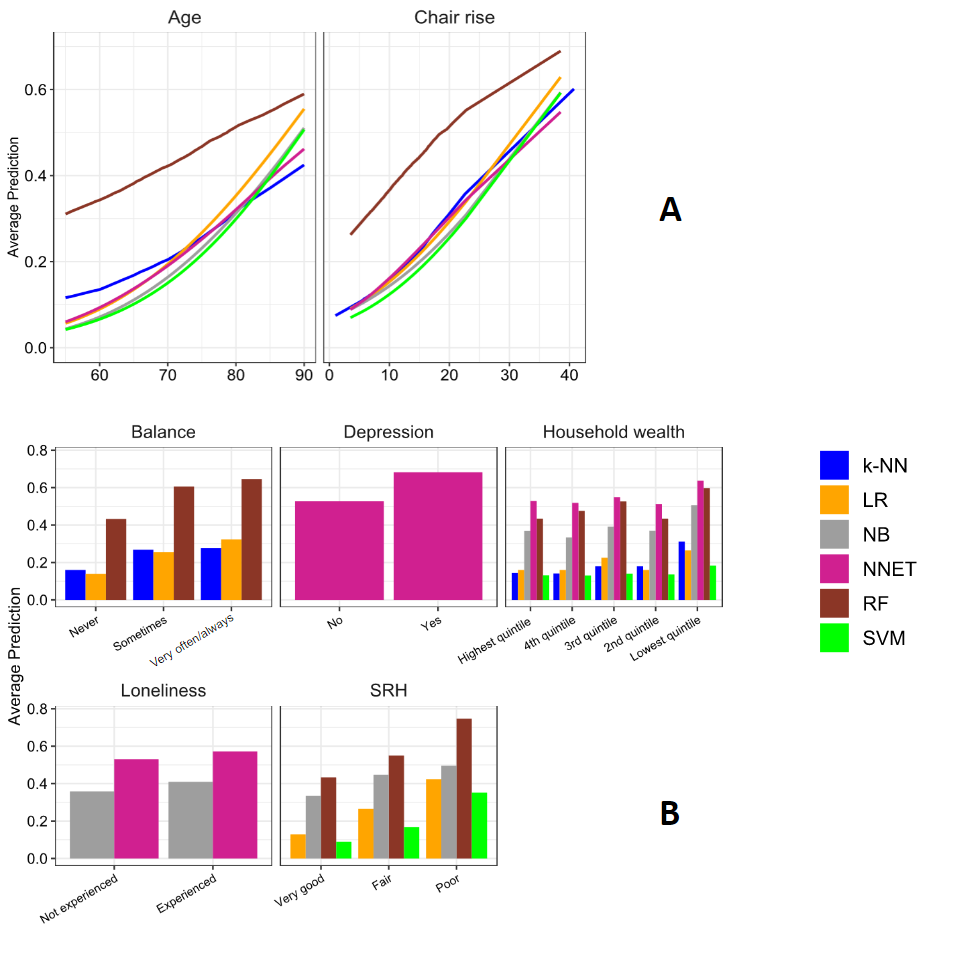


**Supplementary Figure 5.** Partial dependence plots with numerical (A) and categorical (B) frailty predictors by ML models trained with the balanced dataset.

Note: LR= Logistic Regression; RF= Random Forest; SVM= Support Vector Machine; NNET= Neural Network; K-NN= K-Nearest Neighbor; NB= Naive Bayes classifier

Supplementary table 1 shows the values of ROC, sensitivity and specificity achieved with each of the models selected for the last stage of the test and the respective parameters used to achieve the best values for each of the metrics. With the models trained with the imbalanced dataset, the ROC values varied from 0.77 to 0.89, while for the same models trained with the balanced dataset, this metric was higher in every case and varied from 0.89 to 0.93. The sensitivity values improved when the models were trained with the balanced dataset. With the exception of the NB model, for which the values of the parameters did not change, the parameters used with the selected models varied when the models were trained with balanced and with imbalanced datasets. The parameters for LR cannot be adjusted. For this model, the stepwise method of predictor variable selection based on the AIC metric was used to achieve the best values of ROC, specificity and sensitivity (Supplementary Table 1).

**Supplementary table 1.** Values of ROC, specificity and sensitivity achieved with each model selected for testing and parameters used

| ***Imbalanced data***  **Models** | **ROC** | **Specificity** | **Sensitivity** | **Parameters used** |
| --- | --- | --- | --- | --- |
| LR | 0.87 | 0.78 | 0.71 | - |
| RF | 0.88 | 0.87 | 0.73 | mtry=2 |
| SVM | 0.77 | 0.82 | 0.68 | sigma = 0.07330215; C = 0.5 |
| NNET | 0.89 | 0.81 | 0.77 | size = 3; decay = 0.1, 0.5, 1.0 |
| K-NN | 0.77 | 0.86 | 0.55 | k=10 |
| NB | 0.85 | 0.78 | 0.60 | fL = 0, usekernel = TRUE; adjust = 1.0 |
| ***Balanced data***  **Models** |  |  |  |  |
| LR | 0.91 | 0.80 | 0.85 | - |
| RF | 0.92 | 0.84 | 0.88 | mtry=3 |
| SVM | 0.93 | 0.79 | 0.88 | sigma = 0.0599491; C = 1.0 |
| NNET | 0.91 | 0.82 | 0.89 | size = 5; decay = 0.1, 0.5, 1.1 |
| K-NN | 0.90 | 0.80 | 0.84 | k = 20 |
| NB | 0.89 | 0.89 | 0.80 | fL = 0, usekernel = TRUE; adjust = 1.0 |

Note: LR= Logistic Regression; RF= Random Forest; SVM= Support Vector Machine; NNET= Neural Network; K-NN= K-Nearest Neighbor; NB= Naive Bayes classifier; and ROC= Receiver-operating characteristic.

^a^The parameters in the LR model cannot be adjusted.

**References**

1. Stekhoven DJ, Bühlmann P. MissForest—non-parametric missing value imputation for mixed-type data. *Bioinformatics*. 2011; 28(1):112-118. doi:10.1093/bioinformatics/btr597

2. Degenhardt F, Seifert S, Szymczak S. Evaluation of variable selection methods for random forests and omics data sets. *Brief Bioinform*. 2019; 20(2):492-503. doi:10.1093/bib/bbx124
